# Improper Foods as a Factor in Infantile Mortality

**Published:** 1906-11-24

**Authors:** Thomas Divine


### Hospital Clinics.

### IMPROPER FOODS AS A FACTOR IN INFANTILE MORTALITY.

By THOMAS DIVINE, M.D., C.M.(Glasg.), D.P.H.(Camb. and Lond.), F.C.S.

Improper feeding is not only in itself a proximate cause of infantile mortality, but also so focalises the factors involved in social status, the concomitants of overcrowding, and the industrial employment of married women that it becomes of interest to indicate its bearing under four heads.

### 1. To what Extent does Artificial (as Largely Presumptive of Improper) Feeding Prevail.

No very comprehensive statistics exist as to the extent to which breast-feeding is carried on, but the following recent figures may be taken as a fairly reliable index of its prevalence under urban conditions of life. It may be here stated that there is reason to believe that in country districts breast-feeding prevails to a much larger extent than in towns.[1] Newsholme,[2] in Brighton, during 1903 and 1904, as the result of a census of 5,358 houses, in house-to-house inspection, found that of 608 infants under one year of age 62.8 per cent. were entirely breast-fed, 13.2 per cent. were fed partly on the breast and partly by hand, and 24 per cent. were entirely hand-fed. Calculated in three monthly age periods, the percentage of breast-fed infants was as follows: Of infants under three months, 82 per cent. were breast-fed; of infants aged three to six months, 63 per cent. were breast-fed; of infants aged six to nine months, 61 per cent. were breast-fed; of infants aged nine to twelve months, 42 per cent. were breast-fed. This indicates, in Brighton, the gradual abandonment of feeding entirely by the breast as age increases. Most significance attaches to a fall from 82 per cent. in the first three months to 63 per cent. in the second. A fall from 61 per cent. at age six to nine months to 42 per cent. at nine to twelve months is of less significance, since the importance of breast-feeding is relatively greater in the first than in the second six months of life. Howarth,[3] from a very complete investigation among all infants born in Derby during the three years November 1900 to November 1903—an investigation including 8,343 infants—found that 63.3 per cent. were breast-fed; 19.5 per cent. were hand-fed; and 17.3 per cent. were breast-fed at first and afterwards wholly hand-fed, or were partly breast-fed and partly hand-fed, from a very early stage of their existence. The numbers on which these percentages are calculated did not include infants dying early and in whom the kind of food given could have no influence, such as deaths from premature birth, congenital defects and malformation, and those occurring before any nourishment was given. Greenwood,[4] from an inquiry in Blackburn in 1904, ascertained the kind of feeding of 1,133 infants under seven months old. The number did not include infants who only lived a day or two. He found that 49.5 per cent. were entirely breast-fed, 17.5 per cent. partly at the breast and partly by hand, and 33 per cent. were wholly fed by hand. It would be interesting to know how many infants in Blackburn at the age period of three to six months were entirely breast-fed, the presumption being that the percentage would be less than 49.5. These figures, it should be noted, are with respect to three urban districts which may be said respectively to represent both the extremes and the mean as regards the employment of married women. Brighton has 18.8 per cent., Derby 9.2 per cent., and Blackburn 37.9 per cent. of married or widowed females engaged in occupation.[5] It is very questionable whether any great advantage accrues to infants partly breast-fed as compared with those entirely hand-fed. Hutchison,[6] a recognised authority on dietetics, is of opinion that less than six months' suckling does not count for much, and, in his experience, if mixed feeding is attempted for more than a few weeks, the mother's milk tends to disappear altogether. Howarth,[7] on the other hand, believes that the risks of hand-feeding are considerably minimised by mixed feeding, and that every mother who is unable to satisfy her infant should be encouraged to continue to feed her child and to supplement any deficiency by means of artificial food.

### 2. The Underlying Conditions which make Artificial Feeding Necessary.

Von Bunge,[8] of Bâle, asserts that the deficiency in maternal milk, which is such an important predisposing cause at the root of infantile mortality, is due to the imbibing of alcohol by the parents. His statistics, drawn from all Europe, show that women with an insufficient supply of milk are usually the daughters of alcoholics. He asserts that if two generations have been alcoholic, the women of the third generation will almost certainly be unable to nurse their children. However that may be, probably few medical men would be found to challenge the statement that breast-feeding in England and Wales is on the decline, at any rate under urban conditions of life. This may arise apparently from sheer physiological inability, from simple *ennui*, or from the force of circumstances, such as the industrial employment of married women of the lower classes, and the social engagements and distractions of those of the upper. The loss of the function would appear to be one of the concomitants of a spurious civilisation. Starr[9] has noted that in his experience, while there are few American women, especially in the well-to-do classes, who do not look upon the duty of nursing their babies as a pleasant one, yet there are many who are completely unable to do so, and a vast number in whom the secretion of milk fails after a few weeks or months of lactation. Holt,[10] speaking specifically of New York, says that in that city at least three children out of every four born in the homes of the well-to-do must be fed at some other font than the maternal breast. Competent observers have testified to the same tendency of affairs in England,[11] the inability being found both in the upper and lower classes of society. No medical man can fail to have had the fact impressed upon him that mothers in the working classes have many incentives to continue breast-feeding if possible. It is easier and more economical than artificial feeding; and the practice is popularly believed to delay the chance of subsequent pregnancy—a circumstance some would appear often anxious to encompass. It should be stated in this connection that Jewish mothers in England, though they tend to concentrate in urban areas, seem peculiarly exempt from this growing disability; moreover, they are more prolific than their Gentile sisters, and this fact has led Hutchison [12] to the opinion that the ability to produce children and the ability to suckle them are in some way related, quite apart from any racial consideration.

Want of sufficient maternal nourishment must interfere with the due discharge of this duty, and to this circumstance is probably due the fact that even breast-fed children occasionally suffer from marasmus. That the factory employment of married women is one of the conditions which make artificial feeding necessary cannot be doubted. I am aware that many mothers in textile districts return to work as soon as the law will permit. In consequence, suckling in many cases is reduced to a matter of weeks, or simply no attempt at all is made to encourage the function of the breast, the mother feeling that for the short time breast-feeding could conveniently be caried on it really is not worth while to begin. The figures already given for Blackburn should be considered in this connection.

### 3. The Qualities of an Artificial Food which make it an Improper Food.

In order to indicate the qualities of an artificial food which make it an improper food one must have regard to the fact that the natural food of the infant is its mother's milk. What constitutes a proper substitute for this, when it, or the milk of some other woman, cannot be obtained, is rather difficult to determine. Unanimity on this subject is hardly to be looked for. It is to be feared that until quite recently the question of what constitutes a proper artificial food for infants has received but scant attention on the part of medical practitioners, and even at the present time it is a matter for regret that the opinion of the profession on this subject should be so divided. It is not contended that any hard-and-fast method of artificial feeding will meet each and every case; but surely on a question such as this one has a right to look for agreement in the elementary principles at least. It is to be hoped, therefore, that the increasing attention which the subject is now receiving from the scientfic physician will establish those principles on a firm physiological basis, for until that is done the profession is in no secure position to rail at the ignorance of untutored mothers. It is not enough to determine which food or method of feeding is better than something worse, or which will not conduce to this or that illness; the physiological requirements of the growing infant must also be satisfied. Important as correct feeding is at all ages, it is not too much to claim, considering the enormous structural development which takes place during the first year of life, that on successful physiological feeding in infancy much of the future physical well-being of the child depends. The principles of all correct physiological feeding of infants must have regard, first and foremost, to the normal composition of human milk as a standard. Human milk varies within wide limits, as the following analyses of different qualities will show [13]:—

*Human Milk.*

| — | Normal. | Poor. | Over rich. | Bad. |
|---|---|---|---|---|
| Fat ... ... | 4.0 | 1.50 | 5.10 | 0.80 |
| Proteids ... ... | 1 to 2 | 2.40 | 3.50 | 4.50 |
| Lactose ... ... | 7.0 | 4.00 | 7.50 | 5.00 |
| Ash ... ... | 0.2 | 0.09 | 0.25 | 0.09 |
| Total solids ... | 12–13 | 7.99 | 16,35 | 10.39 |
| Water ... ... | 88–87 | 92.01 | 83.65 | 89.61 |

In order to provide a food approaching the normal composition of human milk, the milk of some other animal must be requisitioned. For all practical purposes one may consider that this will be the milk of the cow, for though that of other animals such as the goat, ass, and mare have been used, the milk of the cow is likely to remain the substitute generally employed. The following analysis of three different milks may be taken as fairly representative of the composition of cows' milk [14]:—

*Cows' Milk.*

| — | Leeds. | Langlois. | Langlois. |
|---|---|---|---|
| Specific gravity ... | 1029.7 | 1031.7 | 1033 |
| Volume of cream ... | — | 10 | 7.7 |
| Fat ... ... ... | 3.75 | 4.0 | 3.34 |
| Lactose ... ... | 4.42 | 5.0 | 4.92 |
| Proteids ... ... | 3.76 | 3.4 | 3.40 |
| Ash ... ... ... | 0.68 | 0.6 | 0.57 |
| Total solids ... | 12.61 | 13.0 | 12.23 |

The following figures, according to Leeds, represent the principal differences between cows' and woman's milk [15]:—

*Cows' and Human Milk Compared.*

| Reaction. | Sound Dairy Milk. | Average Woman's Milk. |
|---|---|---|
| | Acid. | Alkaline. |
| Specific gravity ... | 1029 | 1031 |
| Fat ... ... ... | 3.75 | 4.13 |
| Lactose ... ... | 4.42 | 7.0 |
| Proteids ... ... | 3.76 | 2.0 |
| Ash ... ... ... | 0.68 | 0.2 |
| Bacteria ... ... | numerous | absent |

It is obvious that cows' milk must be modified before it can approach the human standard. Not only are the proteids in excess, but the difference between those of cows' milk and woman's milk is considerable. In both cases the proteid consists of caseinogen and lactalbumen, the former in cows' milk being in much larger quantities than in woman's It is the excess of this caseinogen in cows' milk which so frequently causes a hard curd to form in the stomach, differing in this respect markedly from the light flocculent precipitate which is characteristic of the curdling of woman's milk. (1) The amount of curd in cows' milk is 3 per cent. (lactalbumen, 0.75 per cent.); in woman's milk it is only 0.63 per cent. (lactalbumen, 1.5 per cent.), so that the amount of curd is nearly five times as great in the former as in the later. (2) Cows' milk contains a smaller quantity of lactose. (3) The fat is about the same. (4) The ash is greater in cows' milk. (5) By the time cows' milk reaches the consumer it is slightly acid, and contains numerous bacteria, while woman's milk is supplied direct to the child and is alkaline and sterile.[16] The first essential in successful artificial feeding is a pure milk supply. Assuming that a milk of reasonable bacterial purity and chemical quality can be obtained, there are at least three essentials for its approximate modification to the normal standard of human milk:—

(1) *Reduction of the Proteids.*—This may be accomplished by dilution with plain (boiled) water or thin barley-water—a procedure which at the same time renders the caseinogen less liable to form a hard curd on coming in contact with the acid gastric juice. Proteid is an all-essential tissue-builder.

(2) *Increase of Fat.*—This is best accomplished by the addition of sound dairy cream free from preservatives. A due proportion of fat is very essential in infant dietary, since a deficiency may be followed by scurvy, rickets, and a depraved nervous system.

(3) *Increase of Lactose.*—This is best done by the addition of pure sugar of milk. Sugar is important as an energy-producer.

Until an artificially-fed infant is seven months old it should be fed on a food modified on the above principles. It is hardly necessary to add that the feedings should be regular as regards time, of definite quantity, and administered at blood-heat; that all milk employed should be carefully stored and handled and used as fresh as possible; and that every care should be taken to keep all bottles and utensils scrupulously clean. Aware of many imperfections in this sketch, I would yet advance it as embodying the *principles*—as illustrated by minimum requirements—of anything claiming to be a proper artificial food—that is, a food which will satisfy the physiological requirements of a growing infant.

As illustrating how the domestic modification of cows' milk may be carried out, and incidentally filling in other particulars, the following table from Starr is of interest[17]:—

*Table of Ingredients, Hours, and Intervals of Feeding, and Total Quantity of Food for a Healthy Artificially-fed Infant from Birth to end of the seventh month.*

| Age. | Cream. | Whey. | Milk. | Milk Sugar. | Salt. | Water. | Hours for feeding. | Intervals. | Total quantity. |
|---|---|---|---|---|---|---|---|---|---|
| During first week | 2 fluid drams | 3 fluid drams | — | 20 grains | — | 3 fluid drams | 5 a.m. to 11 p.m. | 2 hours | 12 fluid ozs. |
| Second to sixth week | 2 fluid drams | — | ½ oz. (fluid) | 20 grains | a pinch | 1 fluid oz. | do. | do. | 17 fluid ozs. |
| Sixth week to end of second month | ½ fluid oz. | — | 10 fluid drams | ½ dram | do. | 10 fluid drams | do. | do. | 30 fluid ozs. |
| Third to sixth month | do. | — | 2 fluid ozs. | 1 dram | do. | 1½ fluid ozs. | 5 a.m. to 10.30 p.m. | 2½ hours | 32 fluid ozs. |
| During sixth and seventh month | do. | — | 3½ fluid ozs. | do. | do. | 2½ fluid ozs. | 7 a.m. to 10 p.m. | 3 hours | 36 fluid ozs. |

One is now in a position to indicate the qualities of an artificial food which render it an improper food: (1) The food may be too rich or too poor in one or other of its constituents, or the proportions may be all wrong; (2) the food may be wrong in kind; (3) the manner of preserving, preparing, and administering food may be at fault. It is no uncommon occurrence to find parents who are anxious to rear a strong child killing him by misguided kindness. Probably as many children suffer from too much food as from too little. The milk, if not sufficiently dilute, contains more caseinogen than the infant can manage, and digestive troubles are apt to follow. Frequently milk is simply diluted with one or two parts of water. Such a milk may be right in proteids, but is sure to be deficient in lactose and fat; and such deficiency is a frequent cause of general malnutrition and of rickets. Simple as is the modification of milk, it is surprising how few mothers really take an intelligent interest in it.

The milk is found not to agree, and recourse is at once had to other substitutes. Among the working classes more especially, condensed milk is looked upon as "babies' food." It is often, at first at least, surprisingly successful. This is especially apt to be the case in a baby who has failed to digest ordinary milk, because, perhaps, of too high a proteid. Condensed milk is poor both in proteid and in fat. It contains abundance of carbohydrate, but this is usually in the form of cane sugar and is on that account objectionable. By and by condensed milk fails; the child is not thriving. A change is almost as a matter of course made to some one or other of the patent proprietary infant foods—too often supported by lying advertisements: "A perfect food for baby." Without specifying any particular food—and their name is legion—it may be safely said that few indeed of them are proper foods for any infant under six months of age, however useful they may be if properly employed at later ages. They for the most part contain an excess of carbohydrate in the form of starch or maltose, and are without exception deficient in fat, and mostly in proteid. The patent food-fed baby is usually large, flabby, and ricketty, and readily falls a victim to acute disease. Among older children—and sometimes among the very young—ignorant mothers pacify them by little tasty bits of anything that is going. Tea is a general favourite with this class of mothers, and infants are indulged with it almost as soon as they can sip.

A want of cleanliness in the storage, preparation, and administration of food is responsible for many infant deaths. Milk is an excellent culture-medium for nearly all pathogenic organisms; and so, if scrupulous cleanliness is not observed, the risk of fatal infantile diarrhœa, especially during the warm months of the year, is very great indeed. This danger is especially great when a tubed feeding-bottle is used. The long rubber tube of the ordinary and popular feeding-bottle is well named the "baby killer." Its use has been made illegal in some of the States of America. It is supposed to be kept clean by the occasional passage of a tube brush or by a soaking in borax and water. The more indifferent do not even make a pretence of precaution; the tube is allowed to remain in a state of putrefaction, and only receives attention when it will not draw. But not even a reasonable amount of care can ensure such a tube being kept even sensibly clean, not to speak of a condition of asepsis. The use of this senseless contrivance is responsible for much suffering among infants. At the least it induces a septic condition of the entire digestive tract and produces chronic diarrhœa. The long-tubed feeding-bottle owes its favour to the force of custom and to the inertia of ignorance. Its special recommendation to the lazy and indifferent is the ease with which such bottles can be laid beside an infant, which can then be left unattended thus to contrive its feeding as best it may.

One other circumstance connected with artificial feeding remains to be noted, and that is the *rôle* of the dummy teat. The dummy teat is mostly confined to artificially-fed infants. The sucking of this teat is ignorantly regarded by many mothers as a panacea for all the fractious moods of infancy, and hence its name, "a comforter." Among the lesser evils of the "comforter" may be mentioned its action in deforming the mouth and nose. The constant sucking forms a vacuum in the back of the mouth leading to a high arching of the palate—a condition easily produced in the softened bones of the artificially-fed. This in turn encroaches on the nasal cavities above, thus leading to respiratory troubles and disturbed sleep. But a more serious and immediate objection to the "comforter" exists on the score of sepsis. It is always moist, and frequently rolls upon the dirty floor, to be forthwith replaced in the child's mouth without any cleansing, or at most after a superficial rub on the nurse's dress or hands. In such manner oral and intestinal sepsis is induced leading to all sorts of digestive and diarrhœal troubles. It is safe to say the "comforter" must exact a heavy toll in the forms of sickness and death.

The question of properly feeding an infant is not always one of cost. It is certainly more expensive to feed a child on condensed milk—if taken at its caloric value—or on patent foods than on cows' milk. Mr. M. Wynter Blyth has recently examined some of the patent foods and condensed milks on the market, and has calculated their energy value. He gives the following table showing the composition and energy value of a sample of condensed milk, three patent foods, and cows' milk as diluted for the use of infants, with which is compared human milk [18]:—

| — | Fat. | Proteids. | Starch. | Other carbohydrates. | Total energy. |
|---|---|---|---|---|---|
| Condensed milk ... | 0.85 | 0.67 | — | 3.67 | 25.34 |
| Hunter's infant food | 1.75 | 2.03 | 1.57 | 2.75 | 42.36 |
| King's do. | 0.46 | 0.89 | 0.40 | 2.83 | 21.21 |
| Neave's do. | 0.86 | 1.21 | 1.87 | 1.28 | 25.87 |
| Cows' milk*... ... | 1.75 | 1.70 | — | 2.37 | 32.90 |
| Human milk ... | 2.1 | 1.5 | — | 4.75 | 53.51 |

* Diluted with equal quantity of water.

### 4. The Relation of Artificial Feeding to Infantile Mortality.

The subject of artificial feeding holds the first place in the consideration of the factors making for a high mortality among infants. A consideration of this subject cannot ignore the influence of parental ignorance, indifference, and neglect, for largely on such influence does the baneful effect of artificial feeding depend. It requires no argument to prove that the only right and proper, as indeed the only natural, method of feeding an infant is by suckling at its mother's breast. Any appeal to statistics in this connection is simply overwhelmingly in favour of natural *versus* artificial feeding.

Infantile diarrhœa and kindred ailments are relatively more frequent in summer and autumn than at other seasons of the year. The effect of artificial feeding would, therefore, be expected to be thrown into relief, so to speak, if the infantile deaths during the former seasons be analysed. This has been done by Hope of Liverpool.[19] The investigation extended over a series of years, and involved inquiries into the circumstances of upwards of one thousand deaths of infants. He found that amongst infants below three months of age, either wholly or partially fed during these seasons on artificial foods, the deaths were 15 times as great as they were amongst an equal number of infants fed upon the breast alone. In other words, out of every 1,000 infants under three months of age, naturally fed upon breast milk alone, 20 died of autumnal choleraic disease; but of the same number of infants at the same age, fed artificially, instead of 20 dying, as many as 300 deaths occurred. Similarly, he found that amongst infants aged 3 to 6 months and 6 to 9 months there was an immensely larger proportion of deaths amongst the artificially fed than amongst the breast fed, although the proportion diminished as the age increased. Newsholme,[20] from an inquiry at Brighton, relative to 87 infants who died from epidemic diarrhœa in 1903 and 1904, found that the deaths of suckled children were about an eighth of what ought to have occurred on the supposition of the average distribution of diarrhœa; the deaths of those suckled and also having farinaceous food were about one-third; the deaths of those having only cows' milk were over four times as great; and the deaths of those having only condensed milk were nine times as many as ought to have occurred on the supposition of average distribution of diarrhœa among infants fed in different ways. Newsholme believes, however, that there is reason to think that these figures understate the superiority of breast fed over artificially fed infants. But even assuming that they do not, and that the Liverpool figures somewhat overstate, the superiority of breast feeding, the broad fact remains that hand-fed infants succumb to summer diarrhœa out of all proportion to those fed at the breast. These are examples of the pronounced operation of artificial feeding, but there is no doubt that its influence on infantile mortality in general is prejudicial at other seasons, and in many other ways.

### Causes of Death in Children per One Thousand.

Howarth, in Derby, analysed the causes of death in three classes of infants, breast-fed, mixed, and hand-fed. The number of children on which the observations were made does not include those dying of premature birth, congenital defects, and malformations; nor were the observations limited to season. The preceding table is an abridgment of his results,[21] showing deaths per 1,000 on number observed.

| — | Breast fed | Mixed. | Hand. |
|---|---|---|---|
| Number of children ... ... | 5,278 | 1,439 | 1,626 |
| Disease. | | | |
| Bronchitis and pneumonia ... | 14.4 | 12.6 | 26.5 |
| Diarrhœal diseases ... ... | 10.0 | 25.1 | 57.9 |
| Marasmus, atrophy, debility | 12.6 | 18.9 | 39.4 |
| Tuberculous diseases ... | 3.6 | 5.6 | 13.6 |
| Convulsions ... ... ... | 15.0 | 20.9 | 25.9 |
| Dentition ... ... ... | 1.4 | 4.9 | 4.4 |
| Zymotics (other than diarrhœa) ... ... ... | 5.4 | 7.7 | 13.0 |
| Totals (including all other diseases)... ... ... | 69.8 | 98.7 | 197.5 |

Three hundred and sixty-eight deaths occurred amongst the breast-fed, 142 amongst those having mixed food, and 831 amongst those entirely hand-fed. Newsholme[22] records, as showing the close connection between methods of feeding and infantile mortality, how that during the sufferings and starvation connected with the siege of Paris in 1870-71, while the general mortality was doubled, that of infants is said to have been reduced by about 40 per cent., owing to mothers being obliged to suckle their infants. The same increase of adult and diminution of infant mortality was seen during the Lancashire cotton famine, when mothers were not at work in the mills.

If anything further were wanted to emphasise the relation of artificial feeding to infantile mortality, it is to be found in the fact that the infantile mortality among Jews in England compares favourably with that of Gentiles living under the same conditions of poverty, overcrowding, and adverse sanitary conditions generally. I am favoured with the following figures bearing on this specific point by Dr. Niven, of Manchester. "The district of Cheetham," writes Dr. Niven, "is largely Jewish, almost entirely so in its poorer portions, though the outer portion contains both well-to-do Jews and well-to-do Christians."[23]

| District. | Deaths of Infants under one year of age per 1,000 births. | | | | |
|---|---|---|---|---|---|
| | 1899. | 1900. | 1901. | 1902. | 1903. |
| City of Manchester ... | 205 | 189 | 198 | 151 | 169 |
| Cheetham sub-district | 104 | 114 | 124 | 97 | 108 |

To my mind the explanation of the marked difference in these infantile mortality rates is that, as a rule, the Jewish mothers suckle their children at the breast, and generally bestow more care on their offspring than do Gentile mothers in a corresponding sphere of life. It was stated by Sir Shirley Murphy,[24] in his evidence before the Physical Deterioration Committee, that in parts of Stepney, where alien immigration had largely taken place, there had occurred an actual decline in the rate of infantile mortality. This obviously indicates that proper feeding in infancy, together with maternal solicitude, is sufficient to counteract in no small measure the influence of unhygienic surroundings.

It would therefore appear that before any substantial reduction of infantile mortality in England and Wales can be hoped for some means must be sought to equip mothers for the task of suckling their children, or if that be impossible, of providing a suitable substitute to a much greater extent than prevails at present.

[1] Minutes of Evidence, Physical Deterioration Committee. [2] Newsholme, "Annual Report on the Health of Brighton," 1904, p. 47. [3] Howarth, "The Influence of Feeding on the Mortality of Infants," Lancet, 1905, vol. ii. p. 211. [4] Greenwood, "Annual Report, Health of Blackburn," 1905, p. 29. [5] Report on the Census, 1901. [6] Hutchinson, Minutes of Evidence, Physical Deterioration Committee, p. 366. [7] Howarth, Lancet, 1905, vol. ii. p. 211. [8] Cited by Carpenter, "Alcohol and Children," Journal of State Medicine, vol. xi., No. 10, p. 605. [9] Starr, "Diseases of Digestive Organs," 1901, p. 29. [10] Holt, quoted by Vincent, "The Nutrition of the Infant," 1904, p. 30. [11] Minutes of Evidence, Physical Deterioration Committee. [12] Hutchinson, Minutes of Evidence, Physical Deterioration Committee, p. 366. [13] After Rotch, Ashby, and Wright, "Diseases of Children," 1899, p. 43. [14] Ashby and Wright, op. cit., p. 45. [15] Ashby and Wright, op. cit., p. 46. [16] Ashby and Wright, op. cit. [17] Starr, "Diseases of the Digestive Organs," 1901, p. 43. [18] From Newsholme's Report on the Health of Brighton, 1904, p. 78. [19] Hope, "Report on the Health of the City of Liverpool," 1903, p. 166. [20] Newsholme, "Report on the Health of Brighton," 1904, p. 47. [21] See Howarth, Lancet, 1905, vol. ii. p. 212. [22] Newsholme, "Vital Statistics," 1899, p. 128. [23] Letter to the writer from Dr. Niven, March 1905. [24] Minutes of Evidence, Inter-Departmental Committee, Physical Deterioration, p. 348.

